# Supplementary material for: Development and validation of the DHIS2 platform for integrating sociomedical data to study wound care outcomes
Source: PLoS One. 2024 Dec 4;19(12):e0308553. doi: 10.1371/journal.pone.0308553 (PMC11616813; doi:10.1371/journal.pone.0308553)
Supplement: S1 File — Supplementary document 1 shows the number of responses for each statement from the participants who participated in the DHIS2 WoundInfo platform review study. (PDF) [file pone.0308553.s001.pdf]

Table 5. Number of participants response to each statement

Regarding	Statement	Strongly Disagree (n)	Disagree (n)	Neither agree nor disagree (n)	Agree (n)	Strongly Agree (n)
**Performance, effort and facilitating conditions**	I would find WoundInfo useful for my job.	0	0	3	5	4
	Using WoundInfo could enable me to accomplish research more quickly.	0	0	4	3	5
	Using WoundInfo could increase my chances of knowledge discovery.	0	0	3	5	4
	My interaction with WoundInfo was clear and understandable.	0	1	3	5	3
	It would be easy for me to become skillful at using WoundInfo.	0	0	4	5	3
	I would find WoundInfo easy to use.	0	3	2	2	5
	I have the resources necessary to use WoundInfo.	0	1	4	4	3
	I have the knowledge necessary to use WoundInfo.	0	2	3	3	4
	WoundInfo is not compatible with other aspects of my work.	1	0	8	3	0
**Attitude, social influencers and behavioral intention**	Using WoundInfo could be fun for exploring new research questions.	0	0	3	3	6
	Clinicians could treat patients in a better way, if WoundInfo analysis was used.	0	0	4	4	4
	Using WoundInfo could motivate me to do better research.	0	0	6	3	3
	People who influence my research/clinical decisions think that I should use WoundInfo.	0	0	6	5	1
	People who are important to me think that I should use WoundInfo.	0	0	6	5	1
	My use of WoundInfo will depend on whether my colleagues use it for their research/clinical practice.	0	1	6	4	1
	I intend to use WoundInfo in the next few months.	1	1	3	5	2
	I predict that I would use WoundInfo in my next research project.	1	1	3	5	2
	I will use WoundInfo only if I am required to use it.	0	1	6	3	2
**Materials provided for the tasks and Survey**	The documentation slides provided clear instructions.	0	2	2	4	4
	The survey questions were clear.	0	0	4	5	3
	I felt there is a disconnect between platform goals and tasks performed	1	2	4	3	2
	I would recommend my colleagues to review the WoundInfo platform.	0	0	2	3	7
