# Supplementary material for: Development and validation of the DHIS2 platform for integrating sociomedical data to study wound care outcomes
Source: PLoS One. 2024 Dec 4;19(12):e0308553. doi: 10.1371/journal.pone.0308553 (PMC11616813; doi:10.1371/journal.pone.0308553)
Supplement: Attachment — Submitted filename: Response to Reviewers.pdf [file pone.0308553.s004.pdf]

March 20, 2024

Editor-in-chief
PLOS One

Subject: Response letter to reviewers

Dear Editor,

Thank you for the opportunity to revise and resubmit our manuscript titled "Development and Validation of the DHIS2 Platform for Integrating Sociomedical Data to Study Wound Care Outcomes" (Manuscript ID: **PONE-D-23-28893)** for further consideration in PLOS One. We greatly appreciate the time and effort from the reviewers and you, in providing valuable feedback to improve our work.

We have carefully addressed all the comments and concerns raised by the reviewers. We have made the necessary revisions throughout the manuscript, which are highlighted for ease of review. The major revisions addressing the comments from the reviewers and the editor are as follows:

Comments from the reviewer:

1. Comment 1: *Maybe an acknowledgement from authors that these were the objectives for this study?*
   Response: We changed the section (pg. 2, paragraph 3) to make it study objective.

2. Comment 2: *Further clarification is required regarding specific clinical trial data used, stage of clinical trial. Measures to address confounding.*
   Response: We changed the paragraph to highlight the clinical studies whose data was imported into the platform as follows: The incorporated clinical studies are registered on ClinicalTrials.gov under the following identifiers: NCT02577120, NCT02581098, NCT03793062, and NCT01101854.

3. Comment 3: *This sounds incomplete.*
   Response: We rephrased the sentence to add more clarity (Section 2.1, pg. 3).

4. Comment 4: *I could not find the predictive results.*
   Response: The detailed forecasting modeling work is beyond the scope of this paper which primarily focuses on the platform and its evaluation.

5. Comment 5: *How was bias controlled?*
   Response: We re-worded the paragraph 3.7 to discuss bias control as follows:

   To control for potential bias, we examined the demographic composition of these zip codes and found that Caucasian patients were primarily located in zip codes 46241, 46221, 46217, 46227, and 46203, while African-American patients were predominantly found in zip code 46222.

   To further investigate potential confounding factors, we analyzed past employment history, current employment, education, and income rates among patients based on their residing zip codes. Our findings indicated that all zip codes with better past and current employment histories also exhibited higher income and education rates. Additionally, zip codes 46254, 46256, 46229, and 46163 demonstrated better current employment, income, and education rates, with the exception of 46254, which did not show higher income levels among patients. By considering these socio-economic factors and their distribution across zip codes, we aimed to minimize bias in our analysis and provide a more comprehensive understanding of the patient population.

6. Comment 6: *Not enough detail about eligibility criteria and methods of selecting the sample. What were the reasons for recruiting only 12 participants for such an important innovation?*
   Response: The expected criteria for the inclusion of participants for the study has been re-written and included in the manuscript (Section 3.8.1, pg. 9). The reason to choose the number of participants is also explained.

7. Comment 7: *It would be helpful to add the results based on their responses to assessed constructs. These results show group differences only.*
   Response: We added the participant responses in a Table in the supplementary document. We also added a Figure representing the participant responses.

8. Comment 8: *This is indicative of future plans, contrary to the statement above, that predictive models were built.*
   Response: We rephrased this line to add more clarity that forecasting models to predict healing trajectories and classification models to predict wound healing status are out of scope for this paper (pg. 11, paragraph 2).

9. Comment 9: *The researchers were able to provide evidence to this claim. A good pilot study.*
   Response: Thank you for the comment. It is indeed a pilot study to judge the feasibility of the WoundInfo platform.

10. Comment 10: *This would be a useful initiative in contexts where standards for data management exist. This would reduce risk of bias.*
    Response: Thank you for the comment.

Response for additional comments from the editor:

11. Thank you for your inquiry regarding the copyright permissions for the figures included in our submitted manuscript. Upon careful review, we have determined that the figures do not require a separate Copyright form. The figures in question were generated using data from the WoundInfo DHIS2 instance, which utilizes the OpenStreetMap layer. OpenStreetMap is licensed under the Open Data Commons Open Database License (ODbL), which allows for the use and reproduction of the data for various purposes, including academic research. The process for creating these figures involved importing the necessary organization units into the DHIS2 instance. The map figures were then generated directly from the built-in map app module within the DHIS2 platform. As a result, the figures are considered original visualizations created by the authors using the available tools and data within the DHIS2 system.

We firmly believe that the revised manuscript has been substantially improved and addresses all the concerns raised during the previous review cycle. The added clarifications, expanded discussions, and additional analyses have strengthened the study's scientific merit and potential impact.

Thank you again for your consideration, and we look forward to hearing from you regarding the outcome of this revised submission.

Sincerely,

Atika Rahman Paddo

Graduate Research Assistant, Indiana University Purdue University Indianapolis
